# A regression based transmission/disequilibrium test for binary traits: the power of joint tests for linkage and association

**DOI:** 10.1186/1471-2156-6-S1-S95

**Published:** 2005-12-30

**Authors:** Emma K Larkin, Kevin C Cartier, Courtney Gray-McGuire

**Affiliations:** 1 Case Western Reserve University, Department of Epidemiology and Biostatistics, Cleveland, OH, 44106, USA

## Abstract

**Background:**

In this analysis we applied a regression based transmission disequilibrium test to the binary trait presence or absence of Kofendred Personality Disorder in the Genetic Analysis Workshop 14 (GAW14) simulated dataset and determined the power and type I error rate of the method at varying map densities and sample sizes. To conduct this transmission disequilibrium test, the logit transformation was applied to a binary outcome and regressed on an indicator variable for the transmitted allele from informative matings. All 100 replicates from chromosomes 1, 3, 5, and 9 for the Aipotu and the combined Aipotu, Karangar, and Danacaa populations were used at densities of 3, 1, and 0.3 cM. Power and type I error were determined by the number of replicates significant at the 0.05 level.

**Results:**

The maximum power to detect linkage and association with the Aipotu population was 93% for chromosome 3 using a 0.3-cM map. For chromosomes 1, 5, and 9 the power was less than 10% at the 3-cM scan and less than 22% for the 0.3-cM map. With the larger sample size, power increased to 38% for chromosome 1, 100% for chromosome 3, 31% for chromosome 5, and 23% for chromosome 9. Type I error was approximately 7%.

**Conclusion:**

The power of this method is highly dependent on the amount of information in a region. This study suggests that single-point methods are not particularly effective in narrowing a fine-mapping region, particularly when using single-nucleotide polymorphism data and when linkage disequilibrium in the region is variable.

## Background

As the characterization of the human genome continues, increasingly dense marker maps are being created using single-nucleotide polymorphisms (SNPs). It has been suggested that the availability of these dense marker maps will make gene mapping by joint linkage and association preferable to tests for either linkage or linkage disequilibrium (LD) alone [1,2] due to the use of only partial information in either of the individual tests.

Assessment of LD has given rise to many different methods, several of which emphasize the utilization of transmission disequilibrium information. The original transmission disequilibrium test (TDT) uses parent-offspring trios, in which at least one parent is heterozygous at the marker locus and the offspring is affected. A χ^2 ^test is then conducted to compare the transmission of an allele in an affected child to the non-transmitted allele [3]. A later extension, the Sib TDT, [4] incorporates the use of information from unaffected siblings when parental data is absent. The TDT has been expanded by Martin et al. [5] to allow for sibships with multiple affected individuals by modeling allele transmission to the affected sibship as a group instead of each sibling separately [5]. George et al. [6] developed a TDT that regresses a quantitative trait on a parentally transmitted allele. The approach allowed for a wide range of pedigree structures, including both concordant affected and discordant sibships, as well as non-independent nuclear families. Additionally, the regression model can simultaneously estimate the magnitude of association with other covariates associated with the trait as well [6].

The TDT and its extensions were designed for assessing linkage to candidate genes that were already known to be associated with the trait of interest. Recently however, transmission-based tests have been applied to samples of non-independent families, becoming a test of linkage alone. The power of such methods has not been thoroughly investigated when there is no prior knowledge of association.

By incorporating the method proposed by George et al. into a regression-based association test for binary traits, we tested the power of a TDT-type test to detect linkage in the presence of LD. We use the Genetic Analysis Workshop 14 (GAW14) simulated data and assess the power and type I error rate.

## Methods

### Sample

The simulated SNP genome scan data (3-cM density) from all 100 replicates of the Aipotu population were used for this analysis, with the authors knowing the simulated parameters. To further explore the magnitude of the impact of the sample size on power, data from the Aipotu, Danacaa, and Karangar populations were pooled and analyzed. We also examined the effect of using denser marker maps on power as well by selecting SNPs with an average spacing of 1 cM and 0.3 cM from the additional genotyping packets.

### Model definition

The outcome trait was presence or absence of Kofendred Personality Disorder (KPD). Covariates A through L from the three classification groups were considered for possible inclusion in the model. Correlations between covariates were examined using FCOR in Statistical Analysis for Genetic Epidemiology (S.A.G.E v. 4.6) in an effort to pare down the model. Because several covariates within each classification were highly correlated (*r *> 0.7), it was not possible to easily identify non-colinear covariates. Thus, we selected one covariate from each of the classification groups for inclusion in the model (C, G, and J; with prevalences 8.5%, 9.0%, and 15.6%, respectively).

### Linkage and association analysis

To conduct the TDT analysis, we defined indicator variables for a transmitted allele (1 if allele of interest was transmitted, 0 if not) for each SNP in offspring of informative matings. In particular, all offspring from a heterozygous × homozygous mating and all homozygous offspring from a heterozygous × heterozygous mating yielded unambiguous identification of the transmitted allele.

Significance of both the transmitted allele indicator variable and covariates C, G, and J in pedigree data was assessed using the ASSOC program in S.A.G.E. This program uses a regression model with a logit link to obtain residuals that approximate normality, while at the same time allowing for the non-independence of family data. For any individual i, with a binary trait y_i _and vector of covariates x_i_, the regression model is of the form:

Logit(P(y_i _= 1)|X_i_)) = β_0 _+ β_1_x_1 _+ β_2_x_2 _+ ... + G_i _+ F_i _+ M_i _+ S_i _+ E_i_,

where G_i _is a random polygenic effect, F_i _is the random nuclear family effect, M_i _is the random marital effect, S_i _is the random sibship effect, and E_i _is the residual individual random effect [7].

In this analysis we included only the residual individual random effect, which was assumed to be normally distributed with mean zero and variance σ_E_^2 ^such that: V [logit(P(y_i _= 1)|X_i_))] = σ_E_^2 ^was estimated. The likelihood was maximized numerically both with and without the specified test covariate (in this case the transmitted indicator variable) and corresponding likelihoods calculated. Standard errors were determined by numerical double differentiation of the log likelihood and *p*-values, based on a Wald test, were calculated for the random, environmental variance and covariate coefficients. *p*-Values are two-sided for all covariate coefficients and one-sided for all variances.

### Type I error and power

To calculate type I error rate, we identified the number of replicates with a *p*-value less than 0.05 in unlinked regions greater than 10 cM away from the simulated disease locus. To determine power, we calculated the number of replicates for which the *p*-value for the marker locus nearest the simulated disease locus was less than 0.05. This was done for chromosomes 1, 3, 5, and 9. Adjustments for multiple comparisons were not performed.

## Results

### Type I error and power

**Figure 1:**
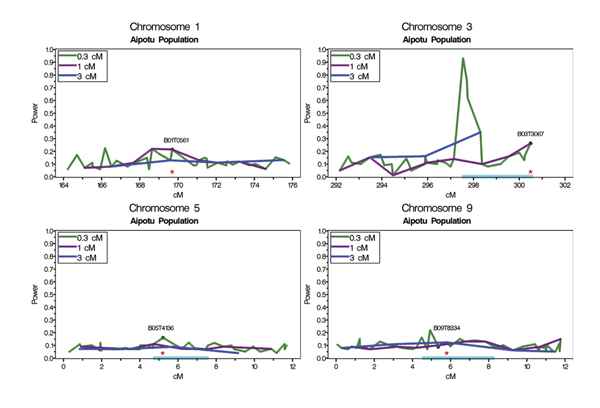
**Power for Aipotu population**. Power of each of the SNPs on chromosomes 1, 3, 5, and 9 at 3 cM, 1 cM, and 0.3 cM density in the Aipotu population. The aqua lines indicate regions of LD; the red stars represent the disease loci; and the black dots represent the markers nearest the disease loci.

In both the Aipotu and the combined populations, the type I error rate was approximately 7%. The power of this method to detect each of the disease loci on chromosomes 1, 3, 5, and 9 was very low when using the Aipotu population alone (Figure 1). On chromosome 1 at a density of 3 cM, the power at the marker nearest the disease locus (C-01-R0052) was 13%. The power was 21% for both the 1-cM and 0.3-cM scan. On chromosome 3, the power to detect linkage in the presence of association was highest (35%) at the marker nearest the disease locus at a density of 3 cM, decreased to 25% at a density of 1 cM, and 26% at 0.3 cM. Power was substantially greater 3 cM away from the disease locus on this same chromosome, reaching 93% using a 0.3-cM scan. Of note, this is the beginning of the region for which LD was simulated. For chromosome 5 the power at the marker nearest the disease locus was 9% at 3-cM density, but increased to 11% and 16% using the finer 1-cM and 0.3-cM scans, respectively. On chromosome 9, 12% power was observed near the disease locus for the 3-cM scan, and for the 1-cM and 3-cM scan, power to detect linkage and association was very similar: 10% and 9%, respectively. Power was greater, in all cases, in regions where LD was said to have been simulated, whether that region included the disease locus or not.

The power was improved in the combined population, but not markedly. On chromosome 1, the power was 17% at the marker nearest the disease locus when using the 3-cM scan and 37% and 38% at the 1-cM and 0.3-cM scan, respectively. At the 3-cM density the power was 63% for the marker nearest the disease gene on chromosome 3 and 42% and 43% for the 1-cM and 0.3-cM scan. For chromosome 5 the power increased from 19% to 31% as the density increased from 3- to 0.3-cM. For chromosome 9, the power was 18% and 23% at 3- and 0.3-cM density, respectively. Again, power increased, in all cases, in regions where LD was said to have been simulated. In fact, on chromosome 3, the method had 100% power at the first marker in the region in which LD was said to be simulated.

## Discussion

This study assesses the power and type I error of a regression-based TDT for binary traits using information from nuclear families. We were able to explore the strength of the method at detecting linkage at varying map densities and sample sizes.

Type I error was stable but slightly inflated (7%), possibly due to transmission distortion [8]. In terms of power, the method performed very well for one of the four simulated disease loci (chromosome 3) at the finest density map of 0.3 cM. The power was above 90% for both the Aipotu and the combined populations at SNP B03T3056, approximately 3 cM away from the disease locus. At the 3-cM density scan the power was much lower, however the marker was closer (approximately 2 cM) to the disease locus than the SNP that produced the maximal power. Other GAW submissions [9-11] that sought to identify association between marker and disease also found the strongest association at SNP B03T3056. We also note that on chromosome 3, the power was higher for the 3-cM scan than the more dense 1-cM map. This is likely due to the fact that 1-cM SNP markers were selected without regard to SNP informativity or proximity to the disease locus. While we would expect that markers closer to the disease locus would yield stronger signals, in this simulation this may not have been the case. Specifically, the strength of the signal appears to be highly dependent on LD in the region. This is certainly more of an issue with single-point methods and we would not expect to see these results if multipoint methods were employed.

Interestingly enough, the power of this method to detect linkage in a region where there was said to be no LD (chromosome 1) was actually higher (21%) than regions on chromosomes 5 and 9 where LD was said to have been simulated (<20%). McCaskie et al. [10] and Song et al. [11] also detected association near the disease marker on chromosome 1 for the Aipotu population in replicate 1, suggesting that some association was present near the disease locus. Our results as well as others' [10-12] further suggest that the LD reported to be on chromosome 5 is weak at best, and hardly detectable. Our results suggest a similarly weak association on chromosome 9, but this was detected by the Song et al. [11].

By using a transmitted allele as the test covariate in the regression model, the sample size was reduced substantially after excluding non-informative individuals (average: 244.4 ± 64.5 individuals, across all SNPs and replicates for Aipotu population). Nevertheless, we were able to explore the impact of increasing sample size by pooling populations. Surprisingly, tripling the average sample size (average: 741.2 ± 202.9 individuals) had a modest impact on power, but the high variability of the sample size makes interpretation of the effects of sample size less straightforward. The gains in power were highest on chromosomes 1 and 3, where presence of a signal was confirmed by other groups with association based tests. For example, power increased to 100% for the 0.3-cM density map on chromosome 3. Power also doubled for the 3-cM density map on chromosome 3 and the 1-cM density map on chromosome 1. Because all populations were simulated with the same disease loci, the modest gain in power due to larger sample size was not likely due to heterogeneity.

To better understand our results, we performed a subsequent analysis, to characterize the amount of LD on chromosomes 1, 3, 5, and 9 by comparing the power at each of the SNPs in the 0.3-cM map to the informativity of those same SNPs (results not shown). There was indeed indication of LD between several of the markers on chromosomes 1 and strong LD on chromosome 3, particularly in regions of strongest signal. These results suggest that our method performed well in the presence of LD. However, even our strongest signal was not very precise. This is likely because it is a single-point method and therefore does not make use of all of the information available in the region.

## Conclusion

While our method performed reasonably well in regions where LD was confirmed, the power was highly dependent on the amount of information in a region, including density of markers and sample size. Certainly, the loss of sample size due to uninformative matings is a weakness of any transmission-based test, and the case in this study as well. Overall, this study suggests that single-point methods, particularly those based on transmitted alleles, are only marginally effective in narrowing a fine-mapping region, particularly when using SNP data containing varying degrees of LD in the region. Further assessment of this method will require detailed information about LD in regions containing the causal locus not available in this dataset.

## Abbreviations

GAW14: Genetic Analysis Workshop 14

KPD: Kofendred Personality Disorder

LD: Linkage disequilibrium

SNP: Single-nucleotide polymorphisms

TDT: Transmission disequilibrium test

## Authors' contributions

EKL participated in the statistical analysis, interpretation of the data and drafting of the manuscript. CG-M conceived of the study, participated in the analysis, and interpretation of the data as well as drafting of the manuscript. KCC performed the statistical analyses and computer programming for this study. All authors read and approved the final manuscript.

## Figures and Tables

**Figure 2:**
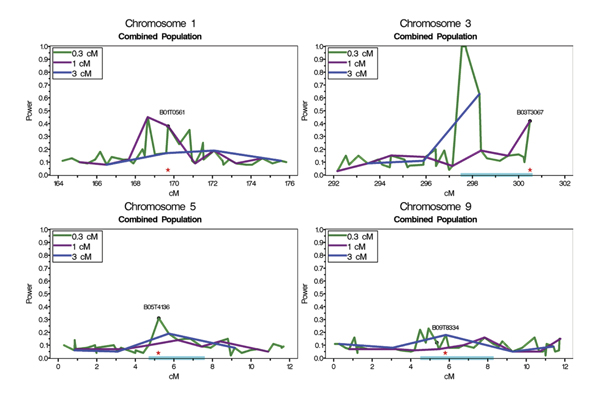
**Power for combined population**. Power of each of the SNPs on chromosomes 1, 3, 5, and 9 at 3 cM, 1 cM, and 0.3 cM density in the combined, Aipotu, Karangar, and Danacaa population. The aqua lines indicate regions of LD; the red stars represent the disease loci; and the black dots represent the markers nearest the disease loci.
